# Practical Notes on Diagnosis and Treatment

**Published:** 1907-12-14

**Authors:** 


### Practical Notes on Diagnosis and Treatment.

**Nocturnal Incontinence.**

Cases of this condition in children and adolescents are sometimes improved by ordering the patient to bed for an hour or so in the middle of the day.

**Quinine in Graves' Disease.**

Quinine sulphate, given in a dose of five to fifteen grains at bedtime, has in some cases of exophthalmic goitre been followed by marked improvement of the symptoms.

**Flatulent Dyspepsia.**

Three drops of oil of cajuput on a piece of sugar or on crumb of bread, taken frequently, is worth all the other antifermentatives put together. It is not only antiseptic but agreeable.—*Dr. Murrell.*

**Laryngismus Stridulus.**

Attacks of laryngeal spasm in infants and young children have on several occasions been found to be associated with elongation of the uvula.

**Endometritis.**

In fungous endometritis and in the metrorrhagia of uterine fibroids potassium iodide is well worthy of trial. It may also be tried in cases of threatened abortion.

**On Full Doses.**

I believe that very often if we were to push our drugs, especially mercury, a little further, in the way our predecessors did seventy years ago, we might sometimes get better results.—*Sir Lauder Brunton.*

